# Capecitabine in hormone-resistant metastatic prostatic carcinoma – a phase II trial

**DOI:** 10.1038/sj.bjc.6601673

**Published:** 2004-03-09

**Authors:** R Morant, J Bernhard, D Dietrich, S Gillessen, M Bonomo, M Borner, J Bauer, T Cerny, C Rochlitz, M Wernli, A Gschwend, S Hanselmann, F Hering, H-P Schmid, for the Swiss Group for Clinical Cancer Research

**Affiliations:** 1 Zentrum für Tumordiagnostik und Prävention (ZeTuP), Rorschacherstrasse 150, St. Gallen CH-9006, Switzerland

**Keywords:** prostatic carcinoma, hormone resistance, capecitabine, prostate-specific antigen (PSA), quality of life, clinical benefit

## Abstract

The objective of the trial is to evaluate the efficacy of capecitabine in patients with metastatic hormone-resistant prostate carcinoma (HRPC), in terms of prostate-specific antigen (PSA) response and clinical benefit (decrease of pain or analgesic score) and its safety profile. In all, 25 patients with HRPC were enrolled on a phase II trial of capecitabine (Xeloda®) at a dose of 1250 mg m^−2^ orally twice daily on days 1–14 every 21 days. The inclusion criteria were PSA serum levels >3 × upper limit of normal, a WHO performance status 0–2, age <85 years and adequate bone marrow, liver and renal function. In patients with grade 2 or higher haematological toxicity on day 1 of the treatment cycle, therapy was first delayed, and then continued at a lower dose. Trial end points were PSA response and clinical benefit defined by quality of life (QL) data and analgesic consumption. The median age of patients was 70 years (range 54–85 years). A median of three cycles of capecitabine was administered (range 1–8). PSA response was observed in three patients (12%, 95% CI 3–31%), with times to tumour progression of 18, 21 and 35 weeks, respectively. In these patients, the response durations were 12, 17 and 32 weeks, respectively. Minor PSA regression was also seen in two further patients. The median time to tumour progression of all patients was 12 weeks (95% CI 9–15 weeks). Haematological toxicity was minor, with leukopenia grade 3 observed in one patient. There were three deaths during trial treatment, respectively, due to sepsis following mucositis and leukopenia, presumed sepsis with mucositis induced by chemotherapy and concomitant radiotherapy and cerebral dysfunction progressing to coma. Hand–foot syndrome grades 2 and 3 were observed in four patients each. Clinical benefit was observed in five patients (20%, CI 7–41%). Based on toxicity data, we recommend a lower starting dose of 1000 mg m^−2^ orally twice daily. While capecitabine has some activity in HRPC, as suggested by observed PSA responses, we conclude that it is not worthwhile to investigate capecitabine monotherapy in a phase III trial. Combinations of capecitabine with other agents, such as vinorelbine or docetaxel, may prove to be more effective.

The role of chemotherapy in patients with hormone-resistant prostate cancer (HRPC) has been re-evaluated in recent years (Goodin *et al*, 2002). The median survival of patients with HRPC remains, however, at 9–12 months despite medical advances and new approaches. Chemotherapy in patients with HRPC has been shown to decrease the serum levels of prostate-specific antigen (PSA), to shrink soft tissue metastases, to improve bone scans and, most importantly, to enhance aspects of quality of life (QL), especially pain (Tannock *et al*, 1996; Fields-Jones *et al*, 1999). Prolongation of survival by chemotherapy, however, has not been demonstrated in phase III trials conducted to date (Tannock *et al*, 1996; Hudes *et al*, 1999; Kantoff *et al*, 1999), although adequately powered randomised trials are now underway comparing mitoxantrone/prednisone with newer treatments including docetaxel alone or in combination with estramustine.

Starting in 1991, the Swiss Group for Clinical Cancer Research (SAKK) conducted four consecutive phase II trials within the framework of a master protocol to evaluate carboplatin (Jungi *et al*, 1998), idarubicin (Schmid *et al*, 1997), gemcitabine (Morant *et al*, 2000) and vinorelbine (Morant *et al*, 2002b), in patients with HRPC. The goal of these studies was to find promising new drugs for further testing in phase III trials. These multicentre trials included similar end points, but during the trials requirements with respect to documentation of response, toxicity, QL and PSA measurements (Schmid *et al*, 2003) have evolved.

The fifth substance to be tested as a part of this programme was capecitabine (Xeloda®), an oral fluoropyrimidine carbamate that delivers 5-FU predominantly to tumour cells. Capecitabine is rapidly and extensively absorbed through the gut as an intact molecule, and is then metabolised to 5-FU in three steps (Budman *et al*, 1998; Miwa *et al*, 1998; Schüller *et al*, 2000). Firstly, it is converted to 5′-deoxy-5-fluorocytidine (5′-DFCR) by carboxylesterase (primarily in the liver). Secondly, it is converted to 5′-deoxy-5-fluorouridine (5′-DFUR) by cytidine deaminase (in tumour cells and in the liver). Finally, it is converted to 5-FU by thymidine phosphorylase (TP), which is significantly more active in tumour tissue than in adjacent healthy tissue (Ishikawa *et al*, 1998). The increasing specificity for tumour cells occurring with each successive conversion step potentially reduces systemic 5-FU exposure while increasing the 5-FU dose within tumour tissue.

Capecitabine is currently approved in over 80 countries worldwide (including Japan, the USA and EU) as monotherapy for the treatment of advanced or metastatic breast cancer patients who have failed previous anthracycline and taxane chemotherapy. In addition, the combination of capecitabine and docetaxel is approved for the treatment of patients with advanced or metastatic breast cancer after failure of cytotoxic chemotherapy including an anthracycline (or when further anthracycline therapy is not possible). Capecitabine is also approved for the first-line treatment of patients with metastatic colorectal cancer.

The objective of the present trial was to evaluate both the efficacy, in terms of PSA response and clinical benefit, and the safety profile of capecitabine in patients with HRPC. The primary end point of the trial was PSA response according to the guidelines by the PSA Working Group (Bubley *et al*, 1999). Quality of life and clinical benefit were secondary end points. In the subset of patients with measurable disease, tumour response following treatment with capecitabine was also documented according to standard WHO criteria.

## PATIENTS AND METHODS

Between March 2000 and June 2001, 25 patients with histologically or cytologically proven metastatic prostatic carcinoma, progressing after orchiectomy or medical castration, were enrolled. Eligibility criteria included an age limit of <85 years, a life expectancy of >12 weeks, a WHO performance status of 0–2 and the absence of known brain metastases. No previous cytotoxic therapy, including estramustine, was allowed. Requirements for laboratory values included a serum PSA of at least three times the upper limit of normal (ULN), leucocytes (WBC) ⩾3.5 × 10^9^ l^−1^ or granulocytes ⩾2 × 10^9^ l^−1^, platelets ⩾100 × 10^9^ l^−1^, haemoglobin ⩾90 g l^−1^, serum creatinine ⩽1.5 × ULN, bilirubin ⩽1.5 × ULN and SGOT ⩽2.5 × ULN. Measurable or nonmeasurable metastatic disease was allowed. Bone metastases alone were considered to be nonmeasurable.

Patients received oral capecitabine at a dose of 1250 mg m^−2^ twice daily for 14 days, repeated every 21 days. Treatment was stopped after a maximum of eight cycles or in the event of tumour progression or severe toxicity. Toxicity was assessed according to the National Cancer Institute of Canada Common Toxicity Criteria (NCIC CTC) and a special toxicity scale for severity of skin toxicity, especially of palms and plants (hand–foot syndrome). Dose reductions and treatment delays were specified in the protocol. In an amendment dated March 2001, the initial capecitabine dose was adapted according to creatinine clearance values.

The trial had a two-stage Gehan design with a sample size of 25 patients in order to estimate the PSA response rate with a standard error of <0.10. If capecitabine had a PSA response rate of less than 20%, then the drug would not be considered worth to be tested in a phase III trial. The trial was designed to be stopped if there were no PSA responses among the first 14 patients. Since two PSA responses were seen, the trial continued beyond the initial 14 patients.

Treatment with antiandrogens, however, had to be stopped at least 1 month before trial enrollment in order not to falsely attribute an antiandrogen withdrawal response (Scher and Kelly, 1993) to the trial drug. Prior radiation therapy was given to 12 patients. Palliative radiation therapy was not allowed within 1 month prior to the start of the study treatment. Previously irradiated metastases were not used for response evaluation.

The trial was approved by the scientific committee of the SAKK, the local ethical committees of participating institutions and regulatory authorities. Written informed consent was obtained from each patient before entering the trial.

The primary end point of the trial was PSA response (Smith *et al*, 1998). In addition, clinical benefit, QL data and, if possible, tumour measurements were recorded.

PSA response was evaluated according to the guidelines for phase II trials in HRPC (Bubley *et al*, 1999). The first response evaluation was performed after two cycles of chemotherapy. A PSA response is defined as a decrease of serum PSA levels by at least 50% compared to baseline and confirmed by a second determination 4 weeks later. Moreover, no increase in the size of pre-existing metastases, no appearance of new lesions and no clinical signs of tumour progression are allowed. Progressive disease (PD) is defined as a confirmed increase of PSA values by at least 25% compared to baseline or nadir levels not reaching response criteria. Time to PSA progression is defined from the first treatment day until the date PSA levels had increased by 50% from the nadir levels for responders or by 25% for patients not reaching a 50% decline of PSA levels. If clinical progression occurred before PSA progression, the date of clinical progression was used. Duration of PSA response is measured from the time at which PSA had declined to ⩽50% to the time when PSA has risen by 50% above the nadir. Time to treatment failure was defined as the time from registration to progression, death or treatment stop due to toxicity or refusal. Confidence intervals for the Kaplan–Meier curves were computed on the log survival scale. PSA levels were determined on day 1 of each treatment cycle.

Pain treatment was not standardised but recorded and classified by the treating physician at registration, on day 8, at the start of each treatment cycle and at treatment failure. A pain treatment score was calculated according to Moore *et al* (1994) for each visit by an independent physician. Standard tablets or capsules of non-narcotic analgesics were assigned 1 point each, and standard doses of narcotic analgesics (e.g., hydromorphone 2 mg, morphine 5 mg, etc.) were assigned two points. These points were totalled for a daily score and averaged into a score assessing the week before the clinical visit.

QL was assessed by the EORTC QLQ-C30 (Aaronson *et al*, 1993) at registration, on day 8, at monthly visits and at treatment failure. This questionnaire has extensively been validated in adults, although not specifically in elderly patients. All scales and single items were transformed according to the EORTC guidelines to range from 0 to 100. A higher score for a functional scale represents a higher level of functioning, a higher score for the global health status/QL scale a better QL, and a higher score for a symptom scale or item a higher level of symptoms or problems. In addition, a global indicator for overall treatment burden (Bernhard *et al*, 2002) and another for coping effort (Hurny *et al*, 1993) were included. These two indicators were transformed accordingly (0–100), with higher scores indicating better QL. All questions referred to the experience during the previous week. Pain (QLQ-C30 items 9+19) was prospectively defined as the primary QL end point, the other measures were used for descriptive purposes only.

Clinical benefit in terms of reduction in patient-rated pain and/or use of analgesics was defined as either a decrease of ⩾2 response categories (corresponding to ⩾33%) in the pain scale (items 9+19) without an increase in analgesics, or a decrease by ⩾50% in analgesics without an increase in pain, measured from baseline for at least two consecutive cycles (i.e. 6 weeks).

For the minority of patients who did not report pain at baseline, we prospectively defined changes in physical functioning and global health/QL (both from QLQ-C30) as benefit criterion as either an improvement of ⩾2 response categories (corresponding to ⩾40%) in physical functioning without any worsening in global health status/QL, or an improvement of ⩾3 response categories (corresponding to ⩾25%) in global health status/QL without any worsening in physical functioning, measured from baseline, for at least two consecutive cycles (i.e., 6 weeks).

We investigated the changes in QL measures from baseline to subsequent time points. Although the transformation of the QLQ-C30 scales results in scores ranging from 0 to 100, these measures are still categorical in nature. Given the small sample, we focused on median values, instead of means. That is, changes that were present in means but not in medians (i.e. median=0) were not considered relevant. Accordingly, we used the exact Wilcoxon signed rank test (based on Hodges–Lehmann (HL) medians), (Lehmann, 1975). All tests were two-sided. No adjustment was made for multiple testing.

## RESULTS

Patient characteristics are shown in Table 1

**Table 1:** Patient characteristics (*n*=25)

	**Category**	**Number**	**%**
Performance status (WHO)	0	8	32
	1	13	52
	2	4	16
Previous radiation therapy	No	13	52
	Yes	12	48
Orchiectomy	No	14	56
	Yes	11	44
LH-RH analogues	No	12	48
	Yes	13	52
Antiandrogens	No	10	40
	Yes	15	60
Measurable disease	No	13	52
	Yes	12	48
Tumor metastases^a^	Bone	24	96
	Lymph nodes	9	36
	Lung	2	8
	Liver	2	8
			
	Median	Range	
Age (years)	70	54–85	
Weight (kg)	80	55–113	
Disease duration since diagnosis (years)	3	0.55–17.9	
PSA (*μ*g/l)	258	15–20400	
Alkaline phosphatase (IU)	325	87–1580	
Creatinine (*μ*mol)	97	58–155	

a Each patient may have more than one tumour localisation.

. The 25 men entered on the trial had a median age of 70 years (range 54–85 years) and generally had a good performance status (84% having a PS of 0 or 1). All patients had documented PD (rising PSA levels and/or new or growing metastases) while on continued androgen ablation: 11 patients had undergone bilateral orchiectomy, 13 had received a luteinising hormone–releasing hormone (LH–RH) agonist, and in one patient LH–RH agonists were erroneously stopped before trial enrolment. A total of 15 patients had been treated during the course of their disease with an androgen receptor blocker either concomitantly or as a second-line hormonal therapy.

Nearly all patients suffered from bone metastases (24 out of 25). In nine patients lymph node metastases were found, in two patients liver metastases and in two lung metastases.

The median number of capecitabine cycles was 3 (range 1–8). The median dose density of capecitabine dropped from 2471 mg m^−2^ during cycle 1 (*n*=25) to 1875 mg m^−2^ during cycle 4 (*n*=11). Dose reductions were necessary in 10 patients, due to hand–foot syndrome (*n*=3), diarrhoea (*n*=2), and in one patient each due to haematological reasons, weight loss, moderate renal function impairment, vomiting and symptoms of vertebral fracture with spinal cord compression (the latter were signs of tumour progression).

Treatment with capecitabine was subjectively well tolerated in the vast majority of patients. The most common clinical treatment-related adverse events (grade 2, 3 or 4, see Table 2

**Table 2:** Grade 2, 3 and 4 adverse events following treatment with capecitabine in 25 patients and 88 treatment cycles

**Toxicity**	**Grade**	**Number of cycles (% of cycles)**	**Number of patients (% of patients)**
Anaemia	2	17 (19%)	9 (36%)
	3	1 (1%)	1 (4%)
Leukopenia	2	4 (5%)	2 (8%)
	3	1 (1%)	1 (4%)
Granulocytopenia	2	1 (1%)	1 (4%)
	3	2 (2%)	2 (8%)
Thrombocytopenia	2	2 (2%)	2 (8%)
Diarrhoea	2	4 (5%)	3 (12%)
	3	1 (1%)	1 (4%)
Neurological	2	4 (5%)	3 (12%)
Nausea	2	10 (11%)	6 (24%)
	3	2 (2%)	2 (8%)
Stomatitis	2	1 (1%)	1 (4%)
	4	1 (1%)	1 (4%)
Hand–foot syndrome	2	9 (10%)	4 (16%)
	3	5 (6%)	4 (16%)

) were nausea (32%), hand–foot syndrome (32%) and diarrhoea (16%). Anaemia was the most commonly observed haematological toxicity (40%), although the majority of cases (36%) were no greater than grade 2 in severity. Other grade 2/3 haematological adverse events were granulocytopenia (12%), leukopenia (12%) and thrombocytopenia (8%), although no grade 4 haematological toxicity was noticed.

Three patients died during the trial: one patient developed mucositis during the first course of chemotherapy and underwent radiation therapy involving the already inflamed oral mucosa. The event was classified as probably related to trial treatment. The patient was admitted to a regional hospital with sepsis, suspected to be related to the injured oropharyngeal mucosa. No agranulocytosis was found. The patient's clinical condition rapidly deteriorated and he died due to a presumed septic shock. The second patient died during the second course after somnolence progressed into coma with a clinical diagnosis of brain metastases or cerebral infarction. Due to the poor clinical status at hospital admission, no further work-up had been done. The third patient died during the first course due to treatment-related toxicity. This 60-year-old obese patient with extensive osseous and hepatic metastases was treated with 5 g of capecitabine daily. He developed mucositis and diarrhoea and was then admitted to the hospital 10 days after starting chemotherapy. Administration of capecitabine was stopped. He was neutropenic and was treated with antibiotics. He succumbed to infection on the tenth day of hospitalisation.

**Figure 1:**
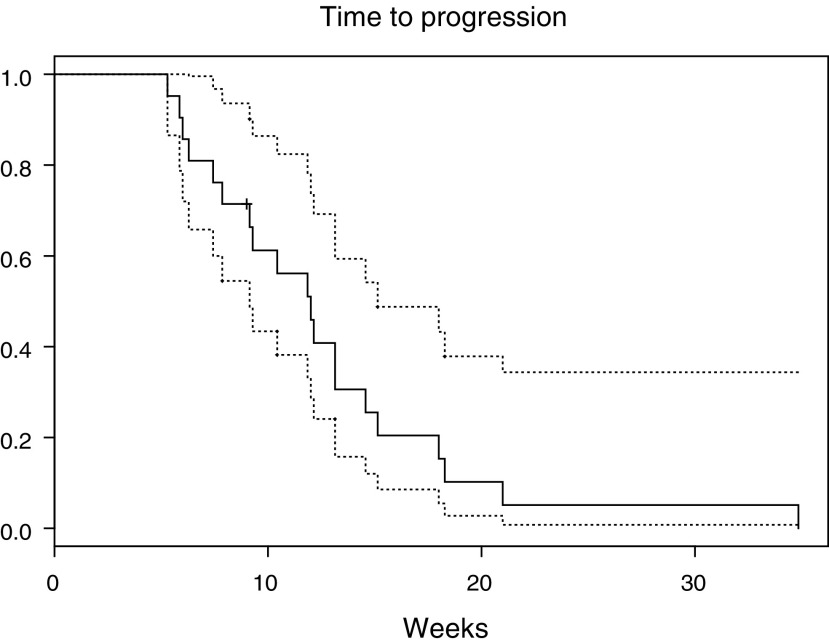
Time to first progression (prostate-specific antigen (PSA) or clinical) measured in weeks with pointwise 95% confidence intervals.

**Figure 2:**
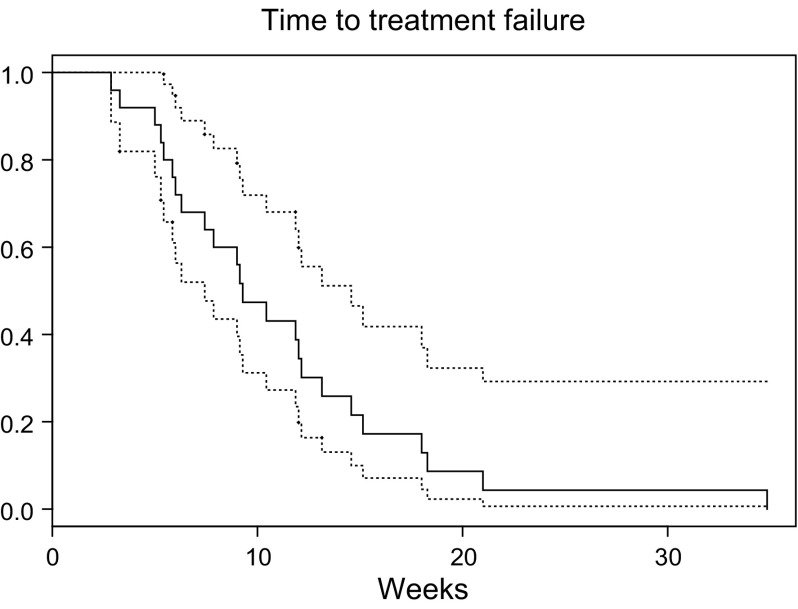
Time to treatment failure (tumour progression, toxicity, death, refusal) in weeks with pointwise 95% confidence intervals.

The median time to tumour progression of the 21 patients completing at least two cycles was 12 weeks (95% CI 9–15 weeks, Figure 1

) and median time to treatment failure of all the 25 patients was 9 weeks (95% CI 7–15 weeks, Figure 2

). Treatment failure was recorded in 20 patients as a result of PD (clinical tumour progression in eight cases, PSA progression in 12 cases), in three patients because of death and in one patient each by patient refusal and unacceptable toxicity.

**Figure 3:**
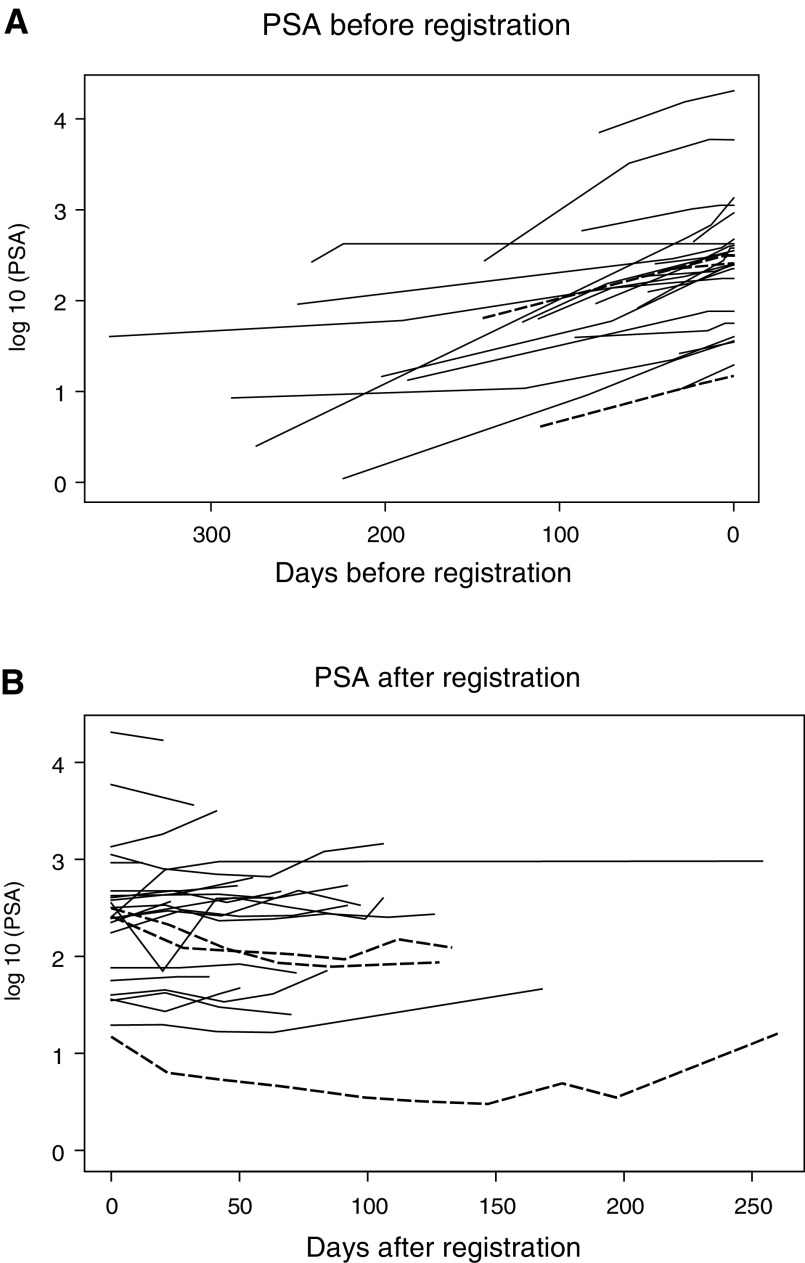
PSA values (**A**) before and (**B**) after trial registration, that is, start of chemotherapy. Each line represents the PSA profile of one patient. The slope of rising PSA levels is less steep after the start of chemotherapy. The dashed lines represent the three cases with a PSA response.

PSA levels of all trial patients before and during treatment with capecitabine are plotted in Figure 3

. A PSA response according to standardised criteria was seen in three patients (12%, 95% CI 3–31%). Response durations and times to tumour progression in these patients were 12, 17 and 32 weeks and 18, 21 and 35 weeks, respectively. Minor reductions in PSA (38 and 41%) were seen in two additional patients. There were no objective tumour responses of measurable disease.

Of all 113 expected pre-failure QL forms, 99 (88%) were received and correctly timed, 24 (96%) at baseline and 75 (85%) during treatment; the submission rate at failure was lower (73%). The reasons for missing QL data were mainly local administrative failure (45%); in 40% of all missing data, there was no reason given. QL data were evaluated up to the beginning of cycle 4; thereafter, too few patients were left on the trial treatment.

At baseline, patients reported a considerably impaired health status/QL (*n*=24, median=50), role functioning (*n*=23, median=66.7), coping (*n*=24, median=58.3) and emotional functioning (*n*=24, median=75). Pain was the most severe symptom (*n*=23, median=50), followed by fatigue (*n*=24, median=33.3) and sleeping disorders (*n*=24, median=33.3).

At day 8 of the first capecitabine cycle, short-term toxicity did not result in worsening in any of the QL measures. Patients reported better emotional functioning (*n*=17, HL median change=12.5, *P*=0.003) and coping (*n*=18, HL median change=8.3, *P*=0.02), and less pain (*n*=17, HL median change=−16.7, *P*=0.02) and sleeping disorders (*n*=18, HL median change=−16.7, *P*=0.05). Besides this early improvement, there was no substantial change over the observation period (up to the beginning of cycle 4).

Physician-rated pain indicated deterioration at the beginning of cycles 3 (*n*=16, median change=−1, *P*=0.04) and 4 (*n*=9, median change=−1, *P*=0.03), when compared to baseline (*n*=25, median=2). The pain treatment score indicated a stable consumption of analgesics (baseline: *n*=24, median=4.6).

In all, 22 patients reported pain at baseline. Five of these (20%) met our criteria for clinical benefit following treatment with capecitabine, and in one case clinical benefit could not be evaluated because of missing data. For at least two consecutive cycles, these patients indicated either a decrease of ⩾2 response categories in the pain scale from baseline without an increase in analgesics, or a decrease by ⩾50% in analgesics without an increase in pain. One of these patients had also a PSA response. The two cases without baseline pain did not meet our criteria for palliation based on physical functioning and global health/QL.

## DISCUSSION

The SAKK has performed five consecutive phase II trials in patients with HRPC, the latest using capecitabine.

Capecitabine has demonstrated consistently high single-agent activity and a favourable safety profile in taxane and anthracycline pretreated metastatic breast cancer (Blum *et al*, 1999, 2001; Fumoleau *et al*, 2001; Talbot *et al*, 2002) and improved overall survival when added to docetaxel in the anthracycline-failure setting (O'Shaughnessy *et al*, 2002). In addition, randomised phase III trials comparing the efficacy and tolerability of 3-weekly intermittent capecitabine with i.v. bolus 5-FU/LV as first-line treatment of advanced colorectal cancer showed that capecitabine was more active than 5-FU/LV in the induction of tumour response, and at least equivalent in terms of time to progression and overall survival (Hoff *et al*, 2001; Van Cutsem *et al*, 2001; Twelves, 2002). In addition to the extensive experience with capecitabine in patients with breast and colorectal cancer, the drug has been investigated in a variety of other cancers, such as pancreatic carcinoma (Hess *et al*, 2003), whereas there is only a single recent case report (El-Rayes *et al*, 2003) describing an impressive response to capecitabine in a patient with HRPC.

Experience with 5-FU in patients with HRPC has been mixed with no responses seen in phase II trials with 5-FU and leucovorin given as bolus injections (Singh *et al*, 1992), high dose infusions every 2 weeks (Atkins *et al*, 1996) or continuous infusion over 5 days (Kuzel *et al*, 1993). However, 5-FU given as a continuous infusion of 300 mg m^−2^ (Hansen *et al*, 1991) showed a significant palliative effect. In prostatic carcinoma, there are significantly higher levels of TP (Mori *et al*, 2000), which is likely to result in higher levels of 5-FU in tumour tissue following administration of capecitabine. The previous benefit of a continuous low dose infusion of 5-FU combined with increased drug accumulation in tumour tissue provided the rationale for this phase II trial.

In this first phase II trial of capecitabine in HRPC, the drug has demonstrated a confirmed PSA response rate of 12% (three of 25 patients) and a mean response duration of 20 weeks (12, 17 and 32 weeks). In addition, two further minor PSA responses were seen. Comparison of PSA values before and after the start of chemotherapy shows that the slope of rising PSA values became less steep in the majority of patients, even if there were no responses (Figure 3). This temporary stabilisation of disease translates into a median time to progression of 12 weeks and a median time to treatment failure of 9 weeks.

Subjective toxicity of capecitabine was mild in the majority of the patients. The most common clinical treatment-related adverse events (grades 2, 3 or 4) were nausea (32%), hand–foot syndrome (32%) and diarrhoea (16%). Anaemia was the most commonly observed haematological toxicity (40%), although no grade 4 haematological toxicity was recorded. Diarrhoea and mucositis were observed in two of the three patients who died during the trial. Considering the observed toxicity and the frequent dose reduction, we recommend a lower starting dose of 1000 mg m^−2^ orally twice daily and to adapt dosages in cases of decreased creatinine clearance.

These results, although not directly comparable, are not substantially different from those of many other phase II trials in HRPC, such as our previous trials with gemcitabine or vinorelbine. However, they do not match the higher response rates reported with docetaxel (Picus and Schultz, 1999) alone or in combinations with estramustine (Petrylak *et al*, 1999) or other agents targeting microtubule functions, such as vinblastine (Hudes *et al*, 1999).

Overall, the QL results indicate that capecitabine was relatively well tolerated. In particular, there was no worsening of QL by short-term toxicity. The improvement in several QL measures over the first 8 days may reflect the beneficial impact of the pain treatment and general support given by the treating physician at baseline, because we do not expect a beneficial effect of chemotherapy on tumour load at this early time point. However, considering the whole observation period, there was no overall improvement in patient- or physician-rated pain. Similarly, the QL measures did not suggest an overall improvement during capecitabine treatment. This is an indication of limited palliation as described in our previous trial of vinorelbine (Morant *et al*, 2002b), which showed a comparable rate of clinical benefit (23%).

In summary, capecitabine monotherapy has proven to be a substance with some activity in patients with HRPC and with generally good tolerability. Careful clinical supervision by an experienced oncologist is, however, necessary, because potentially life-threatening complications, such as stomatitis, leukopenia or diarrhoea may develop, necessitating treatment interruption or dose reduction.

The activity of capecitabine in this patient population is limited concerning both PSA response and clinical benefit. Hence, capecitabine cannot be recommended as a single first-line drug in patients with metastatic HRPC, and we do not think that it is worthwhile to investigate capecitabine monotherapy in a phase III trial. Whether combinations of capecitabine with noncytotoxic agents or other cytostatic drugs, such as docetaxel or vinorelbine, which are combination regimens with proven efficacy in breast carcinoma, will also be useful in this patient population, will require further investigation.

## References

[bib1] Aaronson NK, Ahmedzai S, Bergman B, Bullinger M, Cull A, Duez NJ, Filiberti A, Flechtner H, Fleishman SB, de Haes JC (1993) The European Organization for Research and Treatment of Cancer QLQ-C30: a quality-of-life instrument for use in international clinical trials in oncology. J Natl Cancer Inst 85: 365–376 8433390 10.1093/jnci/85.5.365

[bib2] Atkins JN, Muss HB, Case LD, Richards II F, Grote T, McFarland J (1996) Leucovorin and high-dose fluorouracil in metastatic prostate cancer. A phase II trial of the Piedmont Oncology Association. Am J Clin Oncol 19: 23–25 8554030 10.1097/00000421-199602000-00005

[bib3] Bernhard J, Maibach R, Thurlimann B, Sessa C, Aapro MS (2002) Patients' estimation of overall treatment burden: why not ask the obvious? J Clin Oncol 20: 65–72 11773155 10.1200/JCO.2002.20.1.65

[bib4] Blum JL, Dieras V, Lo Russo PM, Horton J, Rutman O, Buzdar A, Osterwalder B (2001) Multicenter, phase II study of oral capecitabine in taxane-pretreated metastatic breast carcinoma patients. Cancer 92: 1759–1768 11745247 10.1002/1097-0142(20011001)92:7<1759::aid-cncr1691>3.0.co;2-a

[bib5] Blum JL, Jones SE, Buzdar AU, LoRusso PM, Kuter I, Vogel C, Osterwalder B, Burger HU, Brown CS, Griffin T (1999) Multicenter phase II study of capecitabine in paclitaxel-refractory metastatic breast cancer. J Clin Oncol 17: 485–493 10080589 10.1200/JCO.1999.17.2.485

[bib6] Bubley GJ, Carducci M, Dahut W, Dawson N, Daliani D, Eisenberger M, Figg WD, Freidlin B, Halabi S, Hudes G, Hussain M, Kaplan R, Myers C, Oh W, Petrylak DP, Reed E, Roth B, Sartor O, Scher H, Simons J, Sinibaldi V, Small EJ, Smith MR, Trump DL, Wilding G (1999) Eligibility and response guidelines for phase II clinical trials in androgen-independent prostate cancer: recommendations from the Prostate-Specific Antigen Working Group. J Clin Oncol 17: 3461–3467 10550143 10.1200/JCO.1999.17.11.3461

[bib7] Budman DR, Meropol NJ, Reigner B, Creaven PJ, Lichtman SM, Berghorn E, Behr J, Gordon RJ, Osterwalder B, Griffin T (1998) Preliminary studies of a novel oral fluoropyrimidine carbamate: capecitabine. J Clin Oncol 16: 1795–1802 9586893 10.1200/JCO.1998.16.5.1795

[bib8] El-Rayes BF, Black CA, Ensley JF (2003) Hormone-refractory prostate cancer responding to capecitabine. Urology 61: 462 10.1016/s0090-4295(02)02248-3 12597973

[bib9] Fields-Jones S, Koletsky A, Wildin G, O'Rourke M, O'Rourke T, Eckardt J, Yates B, McGuirt C, Burris III HA (1999) Improvements in clinical benefit with vinorelbine in the treatment of hormone-refractory prostate cancer. Ann Oncol 10: 1307–1310 10631457 10.1023/a:1008315106697

[bib10] Fumoleau P, Largillier R, Trillet-Lenoir V (2001) Phase II study of capecitabine (Xeloda®) in pts with advanced breast cancer (ABC), previously treated with anthracyclines and taxanes. Breast Cancer Res Treat 69: 285 (abstract 435)

[bib11] Goodin S, Rao K, DiPaolo R (2002) State-of-the-art treatment of metastatic hormone-refractory prostate cancer. Oncologist 7: 360–370 12185298 10.1634/theoncologist.7-4-360

[bib12] Hansen R, Moynihan T, Beatty P, Quebbeman E, Libnoch J, Schulte W, Anderson T (1991) Continuous systemic 5-fluorouracil infusion in refractory prostatic cancer. Urology 37: 358–361 2014602 10.1016/0090-4295(91)80266-a

[bib13] Hess V, Salzberg M, Borner M, Morant R, Roth AD, Ludwig C, Herrmann R (2003) Combining capecitabine and gemcitabine in patients with advanced pancreatic carcinoma: a phase I/II trial. J Clin Oncol 21: 66–68 12506172 10.1200/JCO.2003.04.029

[bib14] Hoff PM, Ansari R, Batist G, Cox J, Kocha W, Kuperminc M, Maroun J, Walde D, Weaver C, Harrison E, Burger HU (2001) Comparison of oral capecitabine versus intravenous fluorouracil plus leucovorin as first-line treatment in 605 patients with metastatic colorectal cancer: results of a randomized phase III study. J Clin Oncol 19: 2282–2292 11304782 10.1200/JCO.2001.19.8.2282

[bib15] Hudes G, Einhorn L, Ross E, Balsham A, Loehrer P, Ramsey H, Sprandio J, Entmacher M, Dugan W, Ansari R, Monaco F, Hanna M, Roth B (1999) Vinblastine versus vinblastine plus oral estramustine phosphate for patients with hormone-refractory prostate cancer: A Hoosier Oncology Group and Fox Chase Network phase III trial. J Clin Oncol 17: 3160–3166 10506613 10.1200/JCO.1999.17.10.3160

[bib16] Hurny C, Bernhard J, Bacchi M, van Wegberg B, Tomamichel M, Spek U, Coates A, Castiglione M, Goldhirsch A, Senn HJ (1993) The Perceived Adjustment to Chronic Illness Scale (PACIS): a global indicator of coping for operable breast cancer patients in clinical trials. Swiss Group for Clinical Cancer Research (SAKK) and the International Breast Cancer Study Group (IBCSG). Support Care Cancer 1: 200–208 8193882 10.1007/BF00366447

[bib17] Ishikawa T, Sekiguchi F, Fukase Y, Sawada N, Ishitsuka H (1998) Positive correlation between the efficacy of capecitabine and doxifluridine and the ratio of thymidine phosphorylase to dihydropyrimidine dehydrogenase activities in tumors in human cancer xenografts. Cancer Res 58: 685–690 9485021

[bib18] Jungi WF, Bernhard J, Hürny C, Hsu Schmitz S, Hanselmann S, Gusset H, Pestalozzi B, Goldhirsch A (1998) Effect of carboplatin on response and palliation in hormone-refractory prostate cancer. Swiss Group for Clinical Cancer Research (SAKK). Support Care Cancer 6: 462–468 9773464 10.1007/s005200050195

[bib19] Kantoff P, Conaway M, Halabi S, Conaway M, Picus J, Kirshner J, Hars V, Trump D, Winer EP, Vogelzang NJ (1999) Hydrocortisone with or without mitoxantrone in men with hormone refractory prostate cancer. J Clin Oncol 17: 2506–2513 10561316 10.1200/JCO.1999.17.8.2506

[bib20] Kuzel TM, Tallman MS, Shevrin D, Braud E, Kilton L, Johnson P, Kozlowski J, Vogelzang NJ, Blough R, Benson III AB (1993) A phase II study of continuous infusion 5-fluorouracil in advanced hormone refractory prostate cancer. An Illinois Cancer Center Study. Cancer 72: 1965–1968 8364875 10.1002/1097-0142(19930915)72:6<1965::aid-cncr2820720629>3.0.co;2-x

[bib21] Lehmann EL (1975) Nonparametrics: Statistical Methods Based on Ranks. San Francisco: Holden-Day

[bib22] Miwa M, Ura M, Nishida M, Sawada N, Ishikawa T, Mori K, Shimma N, Umeda I, Ishitsuka H (1998) Design of a novel oral fluoropyrimidine carbamate, capecitabine, which generates 5-fluorouracil selectively in tumours by enzymes concentrated in human liver and cancer tissue. Eur J Cancer 34: 1274–1281 9849491 10.1016/s0959-8049(98)00058-6

[bib23] Moore MJ, Osoba D, Murphy K, Tannock IF, Armitage A, Findlay B, Coppin C, Neville A, Venner P, Wilson J (1994) Use of palliative end points to evaluate the effects of mitoxantrone and low-dose prednisone in patients with hormonally resistant prostate cancer. J Clin Oncol 12: 689–694 7512127 10.1200/JCO.1994.12.4.689

[bib24] Morant R, Bernhard J, Maibach R, Borner M, Fey MF, Thurlimann B, Jacky E, Trinkler F, Bauer J, Zulian G, Hanselmann S, Hurny C, Hering F (2000) Response and palliation in a phase II trial of gemcitabine in hormone-refractory metastatic prostatic carcinoma. Swiss Group for Clinical Cancer Research (SAKK). Ann Oncol 11: 183–188 10761753 10.1023/a:1008332724977

[bib25] Morant R, Dietrich D, Gillessen S, Bonomo M, Borner M, Bauer J, Cerny T, Rochlitz C, Wernli M, Gschwend A, Hanselmann S, Hering F, Bernhard J, for the Swiss Group for Clinical Cancer Research (2002a) Capecitabine in hormone-refractory metastatic prostate cancer: a phase II trial of the SAKK. Proc Am Soc Clin Oncol 21: A2442

[bib26] Morant R, Hsu Schmitz SF, Bernhard J, Thurlimann B, Borner M, Wernli M, Egli F, Forrer P, Streit A, Jacky E, Hanselmann S, Bauer J, Hering F, Schmid HP (2002b) Vinorelbine in androgen-independent metastatic prostatic carcinoma – a phase II study. Eur J Cancer 38: 1626–1632 12142053 10.1016/s0959-8049(02)00145-4

[bib27] Mori K, Hasegawa M, Nishida M, Toma H, Fukuda M, Kubota T, Nagasue N, Yamana H, Hirakawa YSCK, Ikeda T, Takasaki K, Oka M, Kameyama M, Toi M, Fujii H, Kitamura M, Murai M, Sasaki H, Ozono S, Makuuchi H, Shimada Y, Onishi Y, Aoyagi S., Mizutani K., Ogawa M, Nakao A, Kinoshita H, Tono T, Imamoto H, Nakashima Y, Manabe T (2000) Expression levels of thymidine phosphorylase and dihydropyrimidine dehydrogenase in various human tumor tissues. Int J Oncol 17: 33–38 10853015 10.3892/ijo.17.1.33

[bib28] O'Shaughnessy J, Miles D, Vukelja S, Moiseyenko V, Ayoub JP, Cervantes G, Fumoleau P, Jones S, Lui WY, Mauriac L, Twelves C, Van Hazel G, Verma S, Leonard R (2002) Superior survival with capecitabine plus docetaxel combination therapy in anthracycline-pretreated patients with advanced breast cancer: phase III trial results. J Clin Oncol 20: 2812–2823 12065558 10.1200/JCO.2002.09.002

[bib30] Petrylak DP, Macarthur R, O'Connor J, Shelton G, Weitzman A, Judge T, England-Owen C, Zuech N, Pfaff C, Newhouse J, Bagiella E, Hetjan D, Sawczuk I, Benson M, Olsson C (1999) Phase I/II studies of docetaxel (Taxotere) combined with estramustine in men with hormone-refractory prostate cancer. Semin Oncol 26: 28–33 10604266

[bib31] Picus J, Schultz M (1999) Docetaxel (Taxotere) as monotherapy in the treatment of hormone-refractory prostate cancer: preliminary results. Semin Oncol 26: 14–18 10604263

[bib32] Scher HI, Kelly WK (1993) Flutamide withdrawal syndrome: its impact on clinical trials in hormone-refractory prostatic carcinoma. J Clin Oncol 11: 1566–1572 7687666 10.1200/JCO.1993.11.8.1566

[bib33] Schmid HP, Maibach R, Bernhard J, Hering F, Hanselmann S, Gusset H, Morant R, Pestalozzi B, Castiglione M (1997) A phase II study of oral idarubicin as a treatment for metastatic hormone-refractory prostate carcinoma with special focus on prostate specific antigen doubling time. Cancer 79: 1703–1709 9128985 10.1002/(sici)1097-0142(19970501)79:9<1703::aid-cncr10>3.0.co;2-1

[bib34] Schmid HP, Morant R, Bernhard J, Maibach R (2003) Prostate specific antigen doubling time as auxiliary end point in hormone refractory prostatic carcinoma. Eur Urol 43: 28–30 12507540 10.1016/s0302-2838(02)00539-0

[bib35] Schüller J, Cassidy J, Dumont E, Roos B, Durston S, Banken L, Utoh M, Mori K, Weidekamm E, Reigner B (2000) Preferential activation of capecitabine in tumor following oral administration to colorectal cancer patients. Cancer Chemother Pharmacol 45: 291–297 10755317 10.1007/s002800050043

[bib36] Singh D, Doroshow JH, Leong L, Margolin K, Akman S, Raschko J, Somlo G, Morgan R, Harrison J, Cho J (1992) Phase II trial of 5-fluorouracil, high-dose leucovorin calcium, and dipyridamole in advanced prostate cancer. J Cancer Res Clin Oncol 119: 117–120 1429827 10.1007/BF01209667 PMC12200751

[bib37] Smith D, Dunn R, Strawderman M, Pienta K (1998) Change in serum prostate-specific antigen as a marker of response to cytotoxic therapy for hormone-refractory prostate cancer. J Clin Oncol 16: 1835–1843 9586898 10.1200/JCO.1998.16.5.1835

[bib38] Talbot DC, Moiseyenko V, Van Belle S, O'Reilly SM, Alba Conejo E, Ackland S, Eisenberg P, Melnychuk D, Pienkowski T, Burger HU, Laws S, Osterwalder B (2002) Randomised, phase II trial comparing oral capecitabine (Xeloda) with paclitaxel in patients with metastatic/advanced breast cancer pretreated with anthracyclines. Br J Cancer 86: 1367–1372 11986765 10.1038/sj.bjc.6600261 PMC2375384

[bib39] Tannock I, Osoba D, Stockler M, Ernst D, Neville A, Moore M, Armitage G, Wilson J, Venner P, Coppin C, Murphy K (1996) Chemotherapy with mitoxantrone plus prednisone or prednisone alone for symptomatic hormone-resistant prostate cancer: a Canadian randomized trial with palliative end points. J Clin Oncol 14: 1756–1764 8656243 10.1200/JCO.1996.14.6.1756

[bib40] Twelves C, on behalf of the Xeloda Colorectal Cancer Group (2002) Capecitabine as first-line treatment in colorectal cancer. Pooled data from two large, phase III trials. Eur J Cancer 38(Suppl 2): 15–20 11841931 10.1016/s0959-8049(01)00415-4

[bib41] Van Cutsem E, Twelves C, Cassidy J, Allman D, Bajetta E, Boyer M, Bugat R, Findlay M, Frings S, Jahn M, McKendrick J, Osterwalder B, Perez-Manga G, Rosso R, Rougier P, Schmiegel WH, Seitz JF, Thompson P, Vieitez JM, Weitzel C, Harper P, Xeloda Colorectal Cancer Study Group (2001) Oral capecitabine compared with intravenous fluorouracil plus leucovorin in patients with metastatic colorectal cancer: results of a large phase III study. J Clin Oncol 19: 4097–4106 11689577 10.1200/JCO.2001.19.21.4097

